# Cardiovascular Risks and Risk Stratification in Inflammatory Joint Diseases: A Cross-Sectional Study

**DOI:** 10.3389/fmed.2022.786776

**Published:** 2022-02-22

**Authors:** Vasyl Yagensky, Michael Schirmer

**Affiliations:** Department of Internal Medicine, Clinic II, Medical University of Innsbruck, Innsbruck, Austria

**Keywords:** quality of health care (MeSH), risk assessment, rheumatology, cardiovascular system, SCORE, systematic coronary risk evaluation, musculoskeletal diseases, inflammatory disease

## Abstract

**Background:**

It is well established that patients with inflammatory joint diseases (IJD) have an increased cardiovascular (CV) mortality and morbidity. According to the 2016 EULAR recommendations on CV risk management, rheumatologists should ensure appropriate management of CV risk in rheumatoid arthritis (RA) and other IJDs. The aim was to assess the CV risk and CV disease in Middle-European patients with IJD.

**Methods:**

A retrospective chart review was performed for CV risk factors and CV disease in outpatients of a rheumatology outpatient clinic. CV risk was assessed according to the 2016 European Guidelines on CV disease prevention and also using 2 other approaches to compare the results with data from Norwegian and Spanish cohorts.

**Results:**

Out of 432 patients, the prevalence of CV disease reached from 8.7% in spondyloarthritis (SpA) and 12.8% in psoriatic arthritis (PsA) to 18.7% in patients with RA. The number of CV risk factors did not differ between patients with RA, SpA, PsA, and non-inflammatory rheumatic disease (NIRD) (with 1.68 ± 0.13, 1.70 ± 0.13, 2.04 ± 0.16, and 1.78 ± 0.34, respectively). CV risk assessment could be performed in 82 patients after exclusion because of missing data and age. Stratification according to ESC guidelines showed low in 50%, moderate in 12.2%, high in 20.7%, and very high CV risk in 17.1% of patients aged between 40 and 65 years. CV risk in the Middle-European patients with IJD was higher than in the German general population (*p* = 0.004), and similar to the Norwegian patients with IJD, although patients with Middle-European PsA were at higher risk than the Norwegian patients (*p* = 0.045). Compared to the Spanish patients, Middle-European patients with IJD were more likely assigned to the high- to a very high-risk group (34.2 vs. 16.2%, *p* < 0.001), especially in RA disease (49.1 vs. 21%, respectively, *p* < 0.001).

**Discussion:**

High prevalence of established CV disease together with high CV risk in patients with IJD urges for increased vigilance for CV risk factors followed by appropriate interaction by the treating physicians. The prospective use of an international CV risk assessment tool will allow not only estimation of the individual CV risk but also provide data for direct comparisons with the general population and other international cohorts.

## Introduction

Cardiovascular (CV) disease has to be considered as the main cause of mortality in the general population. According to the 2017 Global Burden of Disease Study, 31.59% of all deaths in the world are attributable to CV disease (1). Therefore, the European Society of Cardiology and other Societies on Cardiovascular Disease Prevention (ESC) make enormous efforts to develop guidelines, leading to reduction of the CV burden both on the individual and the population levels (2). As systematic screening for CV risks may result only in improvements of risk factors but has an effect on CV disease outcomes on its own, opportunistic screening for CV risk factors is recommended, although a beneficial effect on a clinical outcome is uncertain (2).

Several studies have shown, that patients with rheumatoid arthritis (RA) have the same risk of an adverse CV event as patients with diabetes mellitus (3, 4). The large Nurse's Health study showed a 2-fold increased myocardial infarction risk in patients with RA compared to those without, even after adjusting for traditional CV risk factors (5). Other inflammatory joint diseases (IJD) like spondylarthritis (SpA) and psoriatic arthritis (PsA) may be also linked to increased CV mortality and morbidity, as a large Canadian retrospective study reported a 36–49% increase in vascular deaths in patients with SpA compared to the general population (6). Although the higher prevalence of traditional CV risk factors in patients with RA plays a major role in higher CV disease prevalence, the association between CV risk factors and a CV outcome seems to be weaker in patients with RA compared to the general population (7), indicating the presence of additional risk factors in IJDs, such as systemic inflammation.

As a consequence, the EULAR (European Alliance of Associations for Rheumatology) recommendations on CV disease risk management propose that risk prediction models should be adapted for patients with RA by a 1.5 multiplication factor, including terms of disease duration, seropositivity, or some extra-articular manifestations if this is not already included in the model (8). Whether the use of the 1.5 multiplication factor increases the percentage of patients initially classified with intermediate risk as having high CV risk is still under debate (9, 10). For clinical practice, CV disease risk assessment is recommended for all patients with IJD at least one time every 5 years and should be reconsidered following major changes in antirheumatic therapy. Indeed, the inflammatory burden of IJD-diseases as a potential risk factor is not incorporated into currently proposed risk prediction models like SCORE (11), SCORE2 (12), and the Framingham tool (13). As a consequence, several works demonstrate that risk assessment tools only provide moderate estimations of the actual risk in patients with inflammatory, when subclinical atherosclerosis screening is used (14) or when further CV outcomes are seen in the follow-up (15).

Data on the use of CV risk assessment in clinical rheumatological routine, however, are rare. Therefore, this study aims to evaluate the prevalence of CV risk factors and CV disease in patients with IJD identified from an Austrian cohort of consecutive rheumatological outpatients and compare the results with two other cohorts from Norway and Spain.

## Materials and Methods

### Study Design

This is a cross-sectional study with data retrospectively obtained from a prospective cohort study in the setting of a secondary/tertiary referral rheumatology clinic. This study is part of the prospective SolutionX project, which recruits consecutive rheumatological outpatients. All patients included in the project between September 27, 2017, and July 5, 2020, and diagnosed with RA, SpA, PsA, or a non-inflammatory rheumatic disease (NIRD) were selected for chart review.

### Chart Review

The chart review was performed from July to August 2020 according to the STROBE recommendations for cross-sectional studies (Supplementary Table 1). Diagnoses are routinely based on the 2010 ACR/EULAR classification criteria for RA (16), the 2010 ASAS criteria for SpA (17), and the 2006 CASPAR criteria for PsA (18), respectively. For the chart review, SpA was defined as ankylosing spondylitis or all other axial and peripheral forms of SpA, except PsA. NIRDs include muscular disbalances, cervical, thoracal, and lumbar syndromes as well as osteoarthritis after exclusion of any other inflammatory rheumatic or hemato-oncologic disease.

Charts were manually screened in the hospital information system (KIS by Cerner, locally adapted). Data from the most complete visit record were used. Missing data were supplemented with data obtained within half a year prior or after the main visit as far as available. The absence of searched comorbidities and medications in the record was interpreted as not diagnosed.

### Cardiovascular Risk Assessment

#### Cardiovascular Risk Factors

Study parameters included patient's and disease's characteristics, as well as CV risk parameters, CV diseases, and CV therapies. CV risk factors included smoking status, diabetes mellitus, and arterial hypertension. Systolic and diastolic blood pressures and body mass index were included as reported; laboratory CV parameters included lipid profiles and HbA1c%. CV diseases included coronary heart disease, myocardial infarction, coronary revascularization, cerebrovascular events with ischemic or hemorrhagic stroke, transient ischemic attack, and/or peripheral artery disease. CV therapies included antihypertensive, lipid lowering, and antiplatelet drugs.

#### Cardiovascular Risk Assessment According to 2016 ESC Recommendation

According to the 2016 ESC guidelines, CV risk assessment was performed using the SCORE calculation for data from patients aged 40 to 65 years, if all parameters were available (gender, age, systolic blood pressure, HDL-c, smoking status) (11). Patients were then stratified into four risk categories in accordance with the abovementioned ESC guidelines on cardiovascular disease prevention. The participants with a background of diabetes or established CV disease as well as those with BP ≥ 180/110 mmHg and total cholesterol > 310 mg/dl were excluded from SCORE application and directly classified, as indicated by ESC guidelines. Risk stratification does not include chronic kidney disease and proteinuria, as data were not available. The stratification criteria are presented in Table 1.

**Table 1:** Cardiovascular 10-year-mortality risk groups based on the 2016 ESC guidelines on cardiovascular disease prevention (11).

**Low-risk**	**SCORE <1%**
Moderate-risk	SCORE is ≥ 1% and <5%.
High-risk	Subjects with: - Markedly elevated single risk factors, in particular cholesterol > 310 mg/dL (e.g., in familial hypercholesterolemia) or BP ≥ 180/110 mmHg. - People with DM without major risk factors. - A calculated SCORE ≥ 5 and <10%.
Very high-risk	Subjects with any of the following: - Documented CV disease includes previous acute coronary syndrome or myocardial infarction, coronary revascularization and other arterial revascularization procedures, stroke and transitory ischemic attack, aortic aneurysm and peripheral artery disease. - DM with a major risk factor such as smoking or marked hypercholesterolemia or marked hypertension. - A calculated SCORE ≥ 10%.

*CV, cardiovascular; DM, diabetes mellitus; SCORE, systematic coronary risk evaluation; BP, blood pressure*.

#### Risk Assessments Based on SCORE 2 and the EULAR-Endorsed 1.5 Multiplicator

According to the recently published 2021 ESC guidelines using the new SCORE 2 algorithm, the SCORE 2 was calculated for all those patients, for whom the original SCORE was calculated to compare the SCORE with the new SCORE 2 (2, 12). The patients were then stratified into three risk categories as proposed by the SCORE 2 protocol (Table 2).

**Table 2:** Age-dependent risk stratification based on the SCORE 2 protocol (12).

	**<50 years**	**50–65 years**
Low- to moderate-risk	<2.5%	<5%
High-risk	2.5 to <7.5%	5 to <10%
Very high-risk	≥7.5%	≥10%

#### Norwegian Approach of Risk Stratification

This approach using the HeartScore version was applied for patients aged 30 to 80 years without established CV disease, diabetes mellitus, antihypertensive drugs, and lipid lowering therapy (19). The HeartScore version of the SCORE with HDL-c for low-risk countries was calculated using a publicly available online tool (11).

#### Spanish Approach of Risk Stratification

This approach with the original 2003 version of the SCORE without HDL was applied for data from patients older than 40 years without established CV disease (20).

#### Comparison of CV Risk With German General Population

Data of the general population from a German cohort were used to estimate the level of CV risk in the patients with IJD (21). According to the German protocol, the patients with established CV disease were excluded.

### Statistical Considerations

All data were anonymized before further analysis using the SPSS program for Windows (version 26).

Continuous data were tested for normal distribution using the Kolmogorov–Smirnov test. Means with SD for the normally distributed and medians with interquartile ranges (IQR) for not normally distributed values were calculated.

The Mann-Whitney U and the Kruskal–Wallis tests were used to compare non-parametric variables between two or more groups, respectively. To compare parametric variables between groups, the Student's *t*-test or a one-way ANOVA test was used as indicated. Differences between nominal variables were analyzed using the chi-square test. For comparison of two non-parametric dependent samples, the Wilcoxon signed-rank test was used.

## Results

### Patient's Demographics and Disease's Characteristics

**Figure 1:**
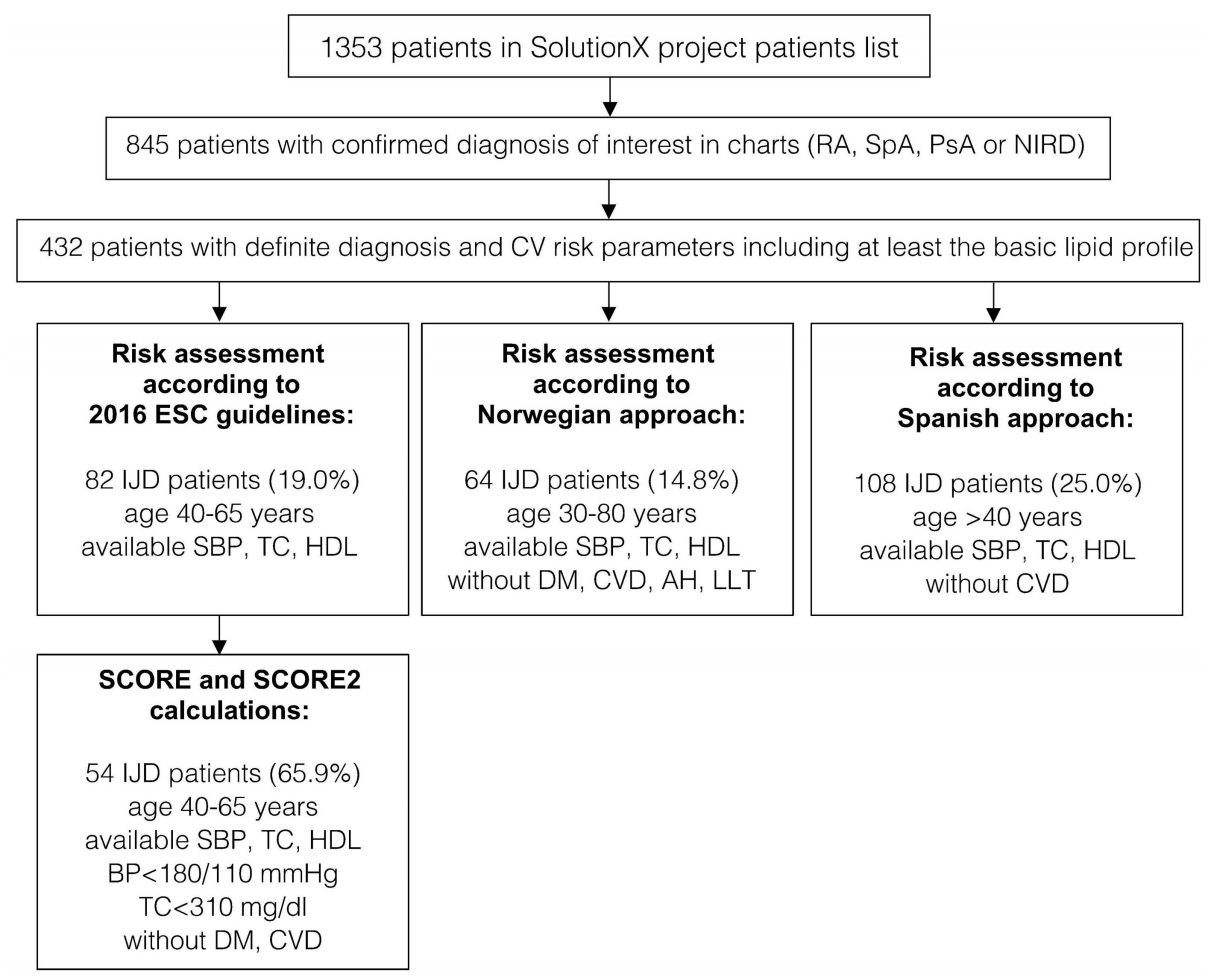
A flow chart of patients' selection and data availability for different analyses (original SCORE and SCORE 2 calculation, risk assessment according to 2016 ESC Guidelines, the Norwegian and the Spanish approaches). RA, rheumatoid arthritis; SpA, spondyloarthritis; PsA, psoriatic arthritis; IJD, inflammatory joint disease (aggregate of RA, SpA, and PsA); NIRD, non-inflammatory rheumatic disease; AH, arterial hypertension; CV, cardiovascular; CVD, cardiovascular disease; DM, diabetes mellitus; ESC, European Society of Cardiology; HDL, high density lipoprotein; LLT, lipid-lowering therapy; TC, total cholesterol; SBP, systolic blood pressure; BP, blood pressure.

Out of the 1,353 patients recruited into the SolutionX project, the most prevalent diagnoses are SpA (*n* = 244, 18%), RA (*n* = 221, 16%), and PsA (*n* = 123, 9%). As shown in Figure 1, charts of these and 404 patients with a non-inflammatory rheumatic diagnosis (NIRD) were screened, and predefined CV risk parameters including at least the lipid profile were available for 432 patients. Out of these, 134 were diagnosed with RA, 115 with SpA, 78 with PsA, and 105 with NIRD.

Patient's and disease's characteristics are summarized in Table 3. Comparative analyses show higher age and more women in the RA group, compared to both other patients with IJDs and NIRD (*p* < 0.001 and <0.007, respectively). The patients with SpA have the longest disease duration among the patients with IJD.

**Table 3:** Patient's and disease's characteristics.

	**RA**	**SpA**	**PsA**	**IJD**	**NIRD**	***p*-value for**	***p*-value**
	***n* = 134 (31%)**	***n* = 115 (26.6%)**	***n* = 78 (18.1%)**	***n* = 327 (75.7%)**	***n* = 105 (24.3%)**	**all groups**	**IJD vs. NIRD**
Median age [years]	64.1 (19.9)	54.2 (16.4)	54.5 (14.1)	57.6 (18.9)	53.9 (21.2)	<0.001^*^	0.025^+^
<45 years [%]	6.7	23.5	17.9	15.3	25.7		
45-60 years [%]	33.6	46.1	47.4	41.3	41.0		
>60 years [%]	59.7	30.4	34.7	43.4	33.3		
Female [%]	76.9	60.0	59.0	66.7	72.4	0.007^o^	0.275^o^
RF+ [%]	66.2						
ACPA+ [%]	66.7						
ANA ≧ 1:80 [%]	44.7						
HLA-B27+ [%]		48.6	20.9				
CRP [mg/dl]	0.27 (0.56)	0.22 (0.39)	0.19 (0.30)	0.22 (0.60)	0.14 (0.26)	<0.001^*^	<0.001^+^
ESR [mm/h]	13 (21)	8 (11)	8 (10)	12 (18)	5 (8)	<0.001^*^	<0.001^+^
Disease duration [years]	11.7 (11)	20.6 (26.7)	9.9 (20.4)	13.2 (15.1)		<0.001^*^	
						***p*** **-value**	***p*** **-value**
						**for IJD groups**	**IJD vs. NIRD**
NSAID, regular [%]	4.5	19.1	17.9	12.8	9.7		<0.001^o^
- On request [%]	36.1	55.7	44.9	45.1	11.7		<0.001^o^
Median CS [mg/day]	0.0 (2.0)	0.0 (1.0)	0.0 (0.9)	0.0 (1.6)	0.0 (0.4)	0.001^*^	<0.001^+^
csDMARD [%]	82.0	19.1	47.7	51.5		<0.001^o^	
tsDMARD [%]	6.0	0.0	12.8	5.5		0.001^o^	
bDMARD [%]	27.8	27.0	20.5	25.8		0.471^o^	

*IJD includes patients diagnosed with RA, SpA, or PsA. ACPA, anti-citrullinated protein antibodies; ANA, antinuclear antibodies; bDMARD, biological disease modifying anti-rheumatic drug; CRP, C-reactive protein; CS, dosis of methylprednisolone; csDMARD, conventional synthetic disease modifying anti-rheumatic drug; ESR, erythrocyte sedimentation rate; IJD, inflammatory joint disease; NIRD, non-inflammatory rheumatic disease; NSAID, non-steroidal anti-inflammatory drug; PsA, psoriatic arthritis; PsO, psoriatic disease; RA, rheumatoid arthritis; RF, rheumatoid factor; SpA, spondylarthritis; tsDMARD, targeted synthetic disease modifying anti-rheumatic drug*.

* *Kruskal–Wallis Test*;

+ *Mann–Whitney-U Test*;

o *chi-squared test. All values are medians with interquartile ranges, if not specified otherwise*.

### Prevalence of Cardiovascular Disease and Risk Factors

Prevalence of CV disease did not differ between disease groups, but, with 18.7%, the patients with RA show the highest prevalence compared to 8.7, 12.8, and 9.5% in patients with SpA, PsA, and NIRD, respectively (Table 4). Prevalence of established CV disease in different IJDs was higher in this cohort than in the Spanish cohort (with 18.7 vs. 10.5%, respectively; *p* = 0.006).

**Table 4:** Cardiovascular disease, cardiovascular risk factors (in alphabetical order) with laboratory findings and current management of patients with RA, SpA, and PsA, grouped as IJD- and compared to patients with NIRD (data given as medians with interquartile ranges, if not specified otherwise).

	**RA**	**SpA**	**PsA**	**IJD**	**NIRD**	***p*-value**
	***n* = 134 (31%)**	***n* = 115 (26.6%)**	***n* = 78 (18.1%)**	***n* = 327 (75.7%)**	***n* = 105 (24.3%)**	**between all groups**
**CV disease [%]**	18.7	8.7	12.8	13.8	9.5	0.075^o^
Acetylsalicylic acid [%]	15.7	10.4	17.9	14.4	11.4	0.371^o^
**Arterial hypertension [%]**	35.1	28.7	37.2	33.3	31.4	0.581^o^
SBP [mmHg]	141 (32)	148 (41)	145 (31)	147 (32)	143 (27)	0.971^*^
DBP [mmHg]	92 (15)	96 (13)	93 (12)	93 (14)	89 (17)	0.371^*^
Antihypertensive therapy [%]	35.8	29.6	33.3	33.0	29.5	0.668^o^
**Diabetes mellitus [%]**	11.2	7.0	9.0	9.2	7.6	0.653^o^
HbA1c%	5.7 (0.6)	5.7 (0.5)	5.6 (0.6)	5.7 (0.5)	5.6 (0.5)	0.446^*^
**Hypercholesterolemia [%]**	48.5	53.0	32.5	46.3	49.5	0.035^o^
Cholesterol [mg/dl] ± SD	196.3 ± 41.5	203.4 ± 44.5	190.6 ± 38.2	197.5 ± 42	200.1 ± 40.5	0.165^A^
**Hypertriglyceridemia [%]**	25.6	30.4	32.1	28.9	29.5	0.742^o^
Triglycerides [mg/dl]	102 (50.8)	122 (72.0)	140 (100.0)	114 (76.3)	132 (82.5)	0.386^*^
LDL-C [mg/dl] ± SD	126.4 ± 38.5	132 ± 41.7	125.2 ± 33.6	128.1 ± 38.6	129.9 ± 36	0.550^A^
HDL-C [mg/dl]	54.5 (19.3)	61(21)	55 (19)	55 (19.3)	69 (19.8)	0.200^*^
Lp (a) [nmol/l]	19 (27.9)	29.5 (59.1)	27.8 (59.6)	19 (51)	19 (43.5)	0.862^*^
Lipid-lowering therapy [%]	18.7	13.0	19.2	16.8	17.1	0.611^o^
**Obesity [%]**	14.4	21.4	25.0	19.6	23.9	0.251^o^
Body-mass index	24.8 (4.5)	25.5 (8)	27.4 (6.9)	25.5 (5.4)	25.0 (2.5)	0.242^*^
**Smoking**						
Current	27.6	25.2	26.9	26.6	27.6	0.973^o^
Previous	11.9	25.2	21.8	19.0	12.4	0.014^o^

*Differences between the groups of patients with IJD and NIRD were not significant; p-values of the differences between all groups of RA, SpA, PsA, and NIRD are indicated in the last column. CV, cardiovascular; DBP, diastolic blood pressure; HbA1c%, glycated hemoglobin; LDL-C, low-density lipoprotein cholesterol; HDL-C, high-density lipoprotein cholesterol; IJD, inflammatory joint disease; Lp (a), lipoprotein (a); NIRD, non-inflammatory rheumatic disease; PsA, psoriatic arthritis; RA, rheumatoid arthritis; SBP, systolic blood pressure; SD, standard deviation; SpA, spondyloarthritis*;

* *Kruskal–Wallis Test*;

o *chi-squared test*;

A *one-way ANOVA*.

The mean number of CV risk factors is 1.77 per patient and does not differ between disease groups, with PsA patients, showing the highest number of CV risk factors (1.68 ± 0.13 for RA, 1.7 ± 0.13 for SpA, 2.04 ± 0.16 for PsA, and 1.78 ± 0.34 for NIRD). Hypercholesterolemia, hypertriglyceridemia, and arterial hypertension are the most prevalent CV risk factors (with 32.5–49.5, 25.6–32.1, and 28.7–37.2% of patients, respectively) (Table 4).

Prevalence of hypercholesterolemia and previous smoking status varies between the groups (*p* = 0.035 and 0.014, respectively). About 32.5% of the patients with PsA show hypercholesterolemia compared to 48.5% of patients with RA, 53% of patients with SpA, and 49.5% of patients with NIRD (*p* = 0.035). Despite the similar prevalence of current smokers in the different groups, the percentage of previous smokers varies between 11.9% of patients with RA, 12.4% of patients with NIRD, 21.8% of patients with PsA, and 25.2% of patients with SpA. There is no difference in the prevalence of diabetes mellitus (with levels of HbA1c%), arterial hypertension (with systolic and diastolic blood pressure), and obesity (with body mass index) between groups as well as between the combined IJD group and the NIRD group. There was only a trend toward higher prevalence of obesity in PsA (with 25 vs. 21.4% in SpA and 14.4% in RA; *p* = 0.179) and a higher BMI in PsA (with 27.4 vs. 25.5% in SpA and 24.8 in RA; *p* = 0.126).

### Assessment of Cardiovascular Risk

Patient's characteristics of the Norwegian and the Spanish cohort are presented in Supplementary Table 2. The Norwegian and the Spanish SpA group included only patients with ankylosing spondylitis. As the Austrian SpA group did not exclusively consist of patients with ankylosing spondylitis, the percentage of women was higher (with 60% compared to 35.1 and 27.1% in the Norwegian and the Spanish groups, respectively) and HLA-B27 positivity was less frequent than in the other cohorts (with 48.6% compared to 85.6 and 76.0% in the Norwegian and the Spanish groups, respectively).

#### Risk Assessment According to 2016 ESC Guidelines

According to the 2016 ESC guidelines, CV risk is assessed in patients aged 40 to 65 years (11). After SCORE calculation and including those patients with established CV disease or diabetes mellitus, the patients are stratified into four CV risk groups (Table 1). Out of the 432 patients, all parameters needed for SCORE calculation were available for 139 patients with IJD. Fifty-seven of them were not within the eligible age of 40 to 65 years and were excluded.

**Figure 2:**
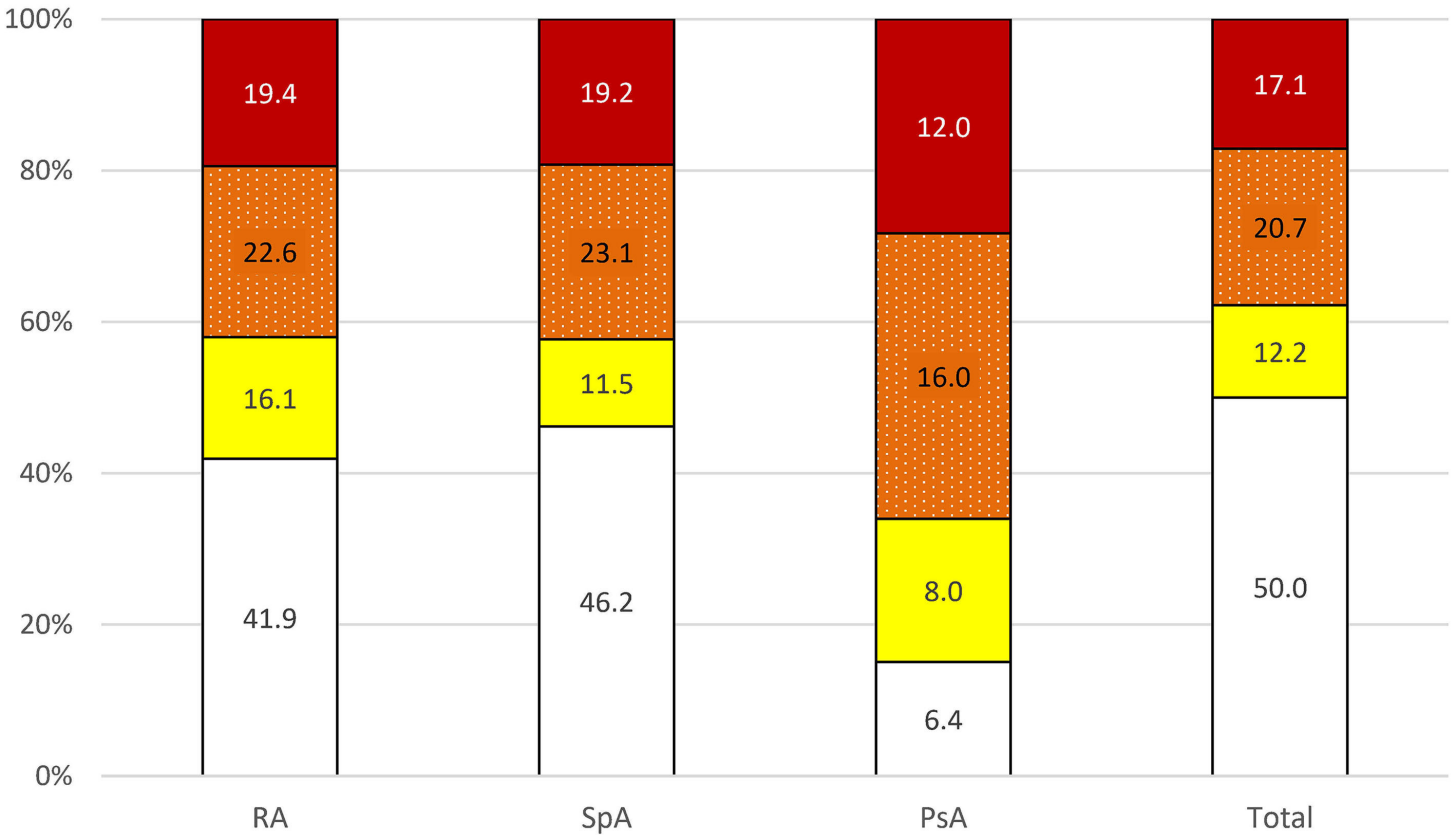
Risk of 10-year cardiovascular mortality in all patients with IJD aged 40 to 65 years according to 2016 ESC guidelines on cardiovascular disease prevention (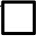 low, 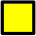 moderate, 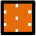 high, and 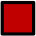 very high risk; data given in percentages). PsA, psoriatic arthritis; RA, rheumatoid arthritis; SpA, spondyloarthritis.

A total of 50% of patients with IJD were classified into the low-risk group, 12.2% into the moderate-, 20.7% into the high-, and 17.1% into the very high-risk groups. As shown in Figure 2, there is no difference in CV risk between the different IJD diseases (*p* = 0.299), although CV risk increases with age (*p* < 0.001), and the median age is highest in the RA group. The median age in the low-risk group is 51.8 (7.4) years, 58.0 (5.5) years in the moderate, 60.0 (9.5) years in the high, and 60.1 (6.2) years in the very high-risk group. Groups do not differ concerning C-reactive protein, erythrocyte sedimentation rate, disease duration, and anti-inflammatory treatment (Supplementary Table 3).

Stratification into the risk groups was based on the calculated SCORE in 65.9% of patients with IJD and based on additional stratification into higher risk categories in 13.4% due to markedly elevated blood pressure (≥180/110 mmHg), in 12.2% due to established CV disease, and in 8.5% of patients with IJD due to diabetes mellitus as comorbidity.

#### Risk Assessments Based on SCORE 2 and the EULAR-Endorsed 1.5 Multiplicator

In those 54 patients, who had been stratified based on the SCORE values, SCORE 2 was calculated for direct comparison of the 2 scores. There was an increase of patients in the high and the very high-risk groups after applying the new SCORE 2 protocol by 40.7 and 7.4%, respectively (*p* < 0.001 using the Wilcoxon-signed-rank test). This can be explained by the definition of SCORE 2, assessing risks for 10-year fatal and non-fatal cardiovascular disease, while the SCORE assesses only risks for 10-year fatal cardiovascular disease. Results are detailed in Supplementary Figure S1.

Application of the 1.5 multiplicators for SCORE values in the RA group as proposed by the EULAR recommendation led to the reclassification of 1 patient (=5%) when using the original SCORE and reclassification of 6 out of the 20 patients (=30%) when using the new SCORE 2.

#### Risk Assessment According to the Norwegian Approach

To compare the retrospective data with Norwegian data, the HeartScore version of the SCORE was calculated for patients with IJD aged 30 to 80 years without established CV disease, diabetes mellitus, lipid-lowering, and antihypertensive therapy (19). Out of 432 patients, parameters for SCORE calculation were available for 139 patients with IJD. Thirteen patients were excluded because of age. SCORE was not calculated for 31 patients because of established CV disease or diabetes mellitus and for 31 other patients because of lipid-lowering or antihypertensive therapy, as they already had an increased CV risk (=62 patients with IJD = 49.2%), compared to 987 Norwegian patients (=39.0%) (*p* < 0.022).

The other 64 out of the 126 eligible patients with IJD (=50.8%) were stratified into two CV risk categories and compared to the Norwegian data (Table 5A). There was no difference in CV risk between these two otherwise CV healthy retrospective disease groups aged 30 to 80 years. Only patients with PsA were at higher risk than Norwegian patients with PsA (*p* = 0.045).

**Table 5:** Cardiovascular risk in patients with IJD (A) without CV disease, diabetes mellitus, lipid-lowering or antihypertensive therapy between the age of 30 and 80 years according to the Norwegian approach (data based only on SCORE values) and in patients with IJD (B) without CV disease older than 40 years according to the Spanish approach (original 2003 SCORE was used).

**CV Risk**	**IJD**	**RA**	**SpA**	**PsA**
**(A)**				
**Middle-European cohort**	*n* = 64	*n* = 29	*n* = 18	*n* = 17
Low to moderate (<5%)	93.8%	93.1%	100%	88.2%
High to very high (≥5%)	6.3%	6.9%	0	11.8%
**Norwegian cohort**	*n* = 2,410	*n* = 1,293	*n* = 613	*n* = 504
Low to moderate (<5%), n	96.8%	95.5%	99.2%	97.0%
High to very high (≥5%)	3.2%	4.5%	0.9%	3.0%
*p*-value	0.184^o^	0.537^o^	0.673^o^	0.045^o^
**(B)**				
**Middle-European cohort**	*n* = 108	*n* = 53	*n* = 29	*n* = 26
Low to moderate (<5%)	65.7%	50.9%	75.9%	84.6%
High to very high (≥5%)	34.2%	49.1%	24.1%	15.3%
**Spanish cohort**	*n* = 1,836	*n* = 693	*n* = 545	*n* = 598
Low to moderate (<5%)	83.8%	79.0%	88.0%	85.6%
High to very high (≥5%)	16.2%	21.0%	12.0%	14.4%
*p*-value	<0.001^o^	<0.001^o^	0.053^o^	0.848^o^

*CV, cardiovascular; IJD, inflammatory joint disease (composite of RA, SpA, and PsA); PsA, psoriatic arthritis; RA, rheumatoid arthritis; SpA, spondyloarthritis; PsA, psoriatic arthritis*;

o *chi-squared test*.

#### Risk Assessment According to the Spanish Approach

According to the Spanish approach, the original 2003 version of the SCORE without HDL was calculated, including patients older than 40 years without established CV disease (20). One hundred eight patients with IJD were included in the stratification according to the Spanish approach.

As shown in Table 5B, our patients with IJD are more likely to be assigned to the high- to the very high-risk group than Spanish patients (34.2 vs. 16.2%; *p* < 0.001). Especially, patients with RA are more often assigned to the high- to very high-risk group compared to the Spanish cohort (49.1 vs. 21.0%, respectively; *p* < 0.001). For RA, there was a trend toward a higher prevalence of hypercholesterolemia than the Spanish patients with RA (43.4 vs. 30.7%; *p* = 0.054). There was also a trend toward a higher prevalence of high to very high risk in the SpA cohort (24.1 vs. 12.0%; *p* = 0.053) but not in the PsA group (15.3 vs. 14.4%; *p* = 0.848).

#### Comparison of CV Risk of Patients With IJD With German General Population

**Figure 3:**
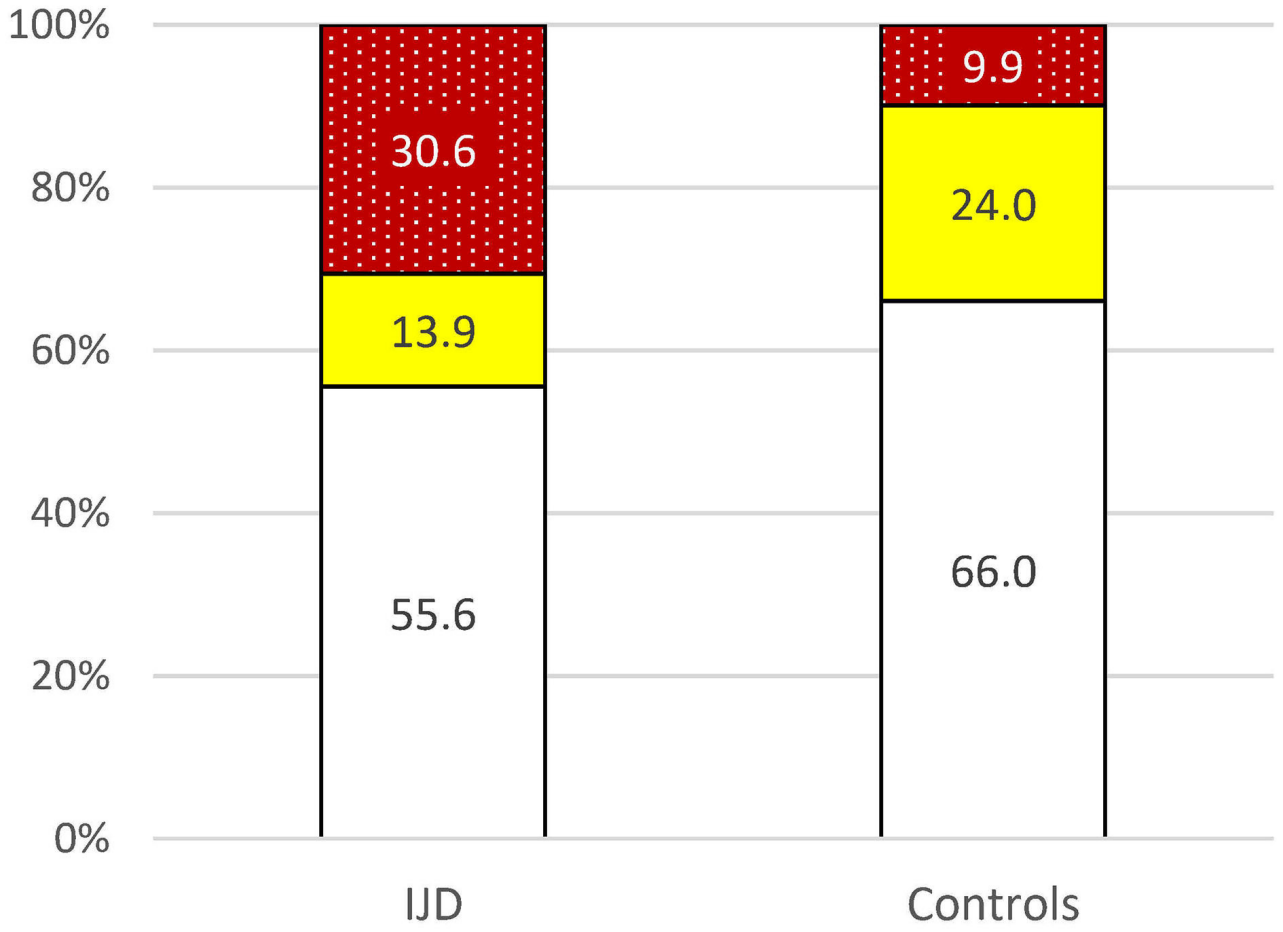
Risk of 10-year cardiovascular mortality in patients with IJD aged 40 to 65 years old and the German control group according to 2016 ESC guidelines on cardiovascular disease prevention but without established CVD for comparison reasons (*p* = 0.004) (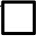 low, 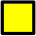 moderate, 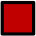 high to very high risk; data given in percentages).

Data of the general population are available for Germany, although patients with established CV disease were excluded (21). The CV risk is higher in the IJD cohort than in the German general population as shown in Figure 3 (*p* = 0.004).

## Discussion

In this cohort, the CV risk was moderate in 12.2%, high in 20.7%, and very high in 17.1% of all patients with IJD aged between 40 and 65 years according to the ESC guidelines (11). This CV risk is higher than the CV risk in the general German population (in persons without established CV disease) (21). Such increased CV mortality is well established for patients with different IJDs (5, 6, 22), but, although the 50% moderate to very high CV risk appears high, it may still underestimate the true CV risk, especially in older patients with IJD (15, 23).

The underlying reason for such underestimation is assumed to be the inflammatory burden of IJD diseases, which is not incorporated into the currently proposed risk prediction models as a potential CV risk factor. Detection of subclinical atherosclerosis could provide more detailed information (14). However, according to the recent 2021 ESC guidelines on CV disease prevention, systematic use of intima-media thickness is not recommended to improve risk assessment due to the lack of methodological standardization and the absence of added value of intima-media thickness in predicting future CV disease events even in the intermediate-risk group (2). In clinical practice, remission or at least low disease activity by the use of potent DMARDs certainly reduces but may not completely abandon the inflammatory burden over time. In this cohort, low disease activity is observed in most patients with normal levels of erythrocyte sedimentation rate and C-reactive protein levels, which was comparable to the Norwegian and the Spanish cohorts (as outlined in Supplementary Table 2).

The EULAR guidelines for the management of CV disease in patients with IJD, therefore, proposed a 1.5-multiplicator for CV risk assessment in patients with RA (8). As this study compared the CV risk in different IJD diseases and multiplicators for patients with SpA and PsA are not available, we applied the risk assessment both with and without the 1.5-multiplicator for patients with RA. Indeed, using the new SCORE 2 algorithm of the ESC group led to the reclassification of patients to a higher risk group in 30% compared to only 5% when using the original SCORE algorithm. Therefore, longitudinal data will be needed to conclude whether the new SCORE 2 algorithm can substitute the 1.5 multiplicators in RA and other inflammatory diseases.

To compare the results with data from different cohorts, this study further assessed the 10-year CV mortality risk both according to the NOCAR project in Norway and the CARMA project in Spain (19, 20). According to the Norwegian approach, CV risk in our patients with IJD aged 30 to 80 years without established CV disease, diabetes mellitus, and lipid lowering and antihypertensive therapy is similar to the Norwegian findings. Only the patients with PsA showed a higher risk than the Norwegian patients with PsA (*p* = 0.045), which could be attributed to the low number of our patients with PsA (*n* = 17). Compared to the Spanish cohort, all our patients with IJD older than 40 years without established CV disease were more likely in the high- to a very high-risk group (with 34.2% vs. 16.2), especially as more patients with RA were assigned to the high- and very high-risk group (49.1 vs. 21%, respectively).

These data fully support the need for lifestyle recommendations, regular CV disease risk management, cautious prescription of non-steroidal anti-rheumatic drugs in RA and PsA, and minimal dosages of corticosteroids as recommended by EULAR (8). Whether and how they are implemented in daily routine care remains open to local organizational concepts. Especially, the benefits of a healthy diet, regular exercise, and smoking cessation should be recommended to all patients with IJD. For this purpose, healthcare teams including nurses may support the rheumatologist and then work in close collaboration with the patients and their families as appropriate (24). Of note, risk assessment is not a one-time event but should be repeated, e.g., every 5 years, although there are no empirical data to guide the length of the intervals (2). Anyhow, for future studies and quality issues, the prospective use of one internationally recommended SCORE will allow both the estimation of the individual CV risk and provide data for benchmarking.

Of note, not only the CV risk but also the prevalence of established CV disease in different IJDs was higher in this cohort than in the Spanish cohort (with 18.7 vs. 10.5%, respectively; *p* = 0.006) (19, 20). Both age and disease duration may explain this finding, as longer disease duration is associated with the development of CV disease in RA (25, 26). Norwegian data were not available for a direct comparison.

Concerning the traditional CV risk factors, lipid abnormalities are often reported in IJD entities (27), with the comparable prevalence of dyslipidemia in different IJDs (19, 20, 28). Hypercholesterolemia was the most frequent CV risk factor in this cohort, but less frequent in patients with PsA than in patients with RA, SpA, and NIRD (with 32.5, 48.5, 53.0, and 49.5%, respectively; *p* = 0.035). This can be partially attributed to slightly albeit not significantly higher use of lipid-lowering medications in these patients. It is well-known that statins have anti-inflammatory effects and can even lower levels of C-reactive protein (29), but levels of C-reactive protein were comparable between patients with IJD, both with and without lipid-lowering therapy (data not shown). For diabetes mellitus, the prevalence was slightly higher in this cohort than reported in other studies. Contrary to an American study (30), our results did not show a higher prevalence of diabetes mellitus in PsA compared to patients with RA (data not shown). Obesity (defined as BMI ≥ 30) is a known risk factor in both CV and rheumatic diseases (31–33), with an increased prevalence of obesity reported for patients with PsA when compared to other patients with IJD (19, 20, 28). In this study, the prevalence of obesity was similar in patients with IJD compared to the general Austrian population (19.6 vs. 20.1%, respectively) (34), and there was only a trend toward a higher prevalence of obesity and a higher BMI in patients with PsA compared to the other patients with IJD.

Concerning arterial hypertension, studies from the literature report varying prevalence between 20 and 40% in patients with IJD, sometimes, even lower than in the control groups (35–37). In line with the literature, the prevalence in this study was similar across the IJD and NIRD disease groups, with the lowest numbers in SpA (with 28.7% in SpA, 35.1% in RA, 37.2% in PsA, and 31.4% in the NIRD group) (19, 20, 28). Smoking is an important risk factor not only for CV but also for the course and, sometimes, even for the treatment responses of disease-modifying antirheumatic drugs in rheumatic diseases (38, 39). In this study, 26.9% of patients are current smokers. These data are comparable to the prevalence of 25.9% in the Austrian general population as published by the WHO (40). Interestingly, the number of ex-smokers was higher in SpA than in the other diseases (*p* = 0.014). Given the varying percentages of CV risk in the different countries, it appears that data cannot be generalized and have to be assessed in each country separately.

Considering the limitations of this study, the retrospective study design with manual data search is certainly inferior to prospective data collection, potentially leading to missing or even biased reporting. Second, the limited number of patients resulted in a lack of statistical power or even unfeasibility of analyses, especially for solid comparisons with patients with NIRD and analyses concerning treatments with CV side effects like non-steroidal anti-inflammatory drugs. Furthermore, a manual not software-supported search of required data in patient's records could produce numerous errors. As real data on the mortality of patients with IJD were missing, a direct comparison between the ESC-derived mortality risk and the use of the 1.5-multiplicator as recommended by EULAR could not be performed. Also, comparisons with the Norwegian and the Spanish cohorts are not adjusted for age as individual data were not available. Complete stratification according to the 2021 ESC guidelines could not be performed in this study, as data for proposed stratification of patients with diabetes mellitus are not routinely available in this center. Contrary to the 2016 guidelines, risk stratification of patients with blood pressure >180/110 mmHg is not detailed in the 2021 version of the ESC guidelines. As controls, the patients with NIRD have selected the best controls we could identify in this setting. For better comparison with the normal population, data were then used from Germany, but we were not able to identify data from the local area or even from Austria as control data.

In view of these data, higher vigilance for CV risk factors followed by appropriate interaction by the treating physicians appears to be justified for all rheumatic patients both with IJD and NIRD to improve the calculated 10-year rate of a major CV event. Routine clinical assessment of the CV risk factors and the use of an international SCORE tool to calculate the CV risk may further support patient's motivation to actively improve their risk of CV disease, e.g., with life-style changes, including dietary efforts and smoking cessation.

## Data Availability Statement

The raw data supporting the conclusions of this article will be made available by the authors, without undue reservation.

## Ethics Statement

The studies involving human participants were reviewed and approved by Ethical Committee of the Medical University Innsbruck (AN 2017-0041 317/4.18). The patients/participants provided their written informed consent to participate in this study.

## Author Contributions

VY and MS substantially contributed to the conception of the study, data acquisition, analysis, and interpretation of the data. All authors agree to be accountable for all aspects of the work in ensuring that questions related to the accuracy or integrity of any part of the work are appropriately investigated and resolved, drafted and revised critically the important intellectual content, and finally approved the manuscript version to be published.

## Funding

The SolutionX project and this study are supported by the Medical University of Innsbruck and the Verein zur Förderung der Hämatologie, Onkologie und Immunologie, Innsbruck.

## Conflict of Interest

The authors declare that the research was conducted in the absence of any commercial or financial relationships that could be construed as a potential conflict of interest.

## Publisher's Note

All claims expressed in this article are solely those of the authors and do not necessarily represent those of their affiliated organizations, or those of the publisher, the editors and the reviewers. Any product that may be evaluated in this article, or claim that may be made by its manufacturer, is not guaranteed or endorsed by the publisher.
