# Supplementary material for: Cardiovascular Risks and Risk Stratification in Inflammatory Joint Diseases: A Cross-Sectional Study
Source: Front Med (Lausanne). 2022 Feb 22;9:786776. doi: 10.3389/fmed.2022.786776 (PMC8904360; doi:10.3389/fmed.2022.786776)
Supplement: Supplementary file 2 [file Table_2.DOCX]

Supplementary table S2. Austrian, Norwegian and Spanish patients’ characteristics according to disease group and study cohort (20,22). The normal German population (n = 3498) included 54.5% female inhabitants (23**)**.

|  | RA | SpA | PsA | IJD |
| --- | --- | --- | --- | --- |
| Number of patients |  |  |  |  |
| Austrian | 134 | 115 | 78 | 327 |
| Norwegian | 1961 | 835 | 721 | 3517 |
| Spanish | 775 | 738 | 721 | 2234 |
| Median age [years] |  |  |  |  |
| Austrian, median | 64.1 (19.9) | 54.2 (16.4) | 54.5 (14.1) | 57.6 (18.9) |
| Norwegian, mean | 59.1±11.2 | 48.4±9.6 | 52.0±10.0 | 55.1±11.6 |
| Spanish, mean | 57.1±12.3 | 48.1±11.7 | 51.8±12.0 | n.p. |
| Female [%] |  |  |  |  |
| Austrian | 76.9 | 60.0 | 59.0 | 66.7 |
| Norwegian | 70.8 | 35.1 | 50.5 | 58.2 |
| Spanish | 75.0 | 27.1 | 45.4 | 49.6 |
| RF+ [%] |  |  |  |  |
| Austrian | 66.2 |  |  |  |
| Norwegian | 66.5 |  |  |  |
| Spanish | 68.1 |  |  |  |
| ACPA+ [%] |  |  |  |  |
| Austrian | 66.7 |  |  |  |
| Norwegian | 77.3 |  |  |  |
| Spanish | 62.2 |  |  |  |
| HLA-B27+ [%] |  |  |  |  |
| Austrian |  | 48.6 | 20.9 |  |
| Norwegian |  | 85.6 |  |  |
| Spanish |  | 76.0 |  |  |
| CRP [mg/dl], median |  |  |  |  |
| Austrian | 0.27 (0.56) | 0.22 (0.39) | 0.19 (0.30) | 0.22 (0.60) |
| Norwegian | 0.30 (0.50) | 0.30 (0.40) | 0.30 (0.40) | 0.30 (0.40) |
| Spanish | 0.31 (0.68) | 0.36 (0.73) | 0.29 (0.47) | n.p. |
| ESR [mm/h], median |  |  |  |  |
| Austrian | 13 (21) | 8 (11) | 8 (10) | 12 (18) |
| Norwegian | 10 (14) | 7 (12) | 8 (12) | 9 (12) |
| Spanish | 17 (20) | 10 (15) | 12 (15) | n.p. |
| Disease duration [years], median |  |  |  |  |
| Austrian | 11.7 (11) | 20.6 (26.7) | 9.9 (20.4) | 13.2 (15.1) |
| Norwegian | 8.3 (11.4) | 10.3 (17.0) | 7.6 (12.2) | 8.4 (12.8) |
| Spanish | 8.0 (11.0) | 15.0 (18.0) | 9.0 (12.0) | n.p. |
| Glucocorticoids [%] |  |  |  |  |
| Austrian, current use | 28.4 | 10.4 | 14.1 | 18.7 |
| Norwegian, current use | 29.3 | 2.0 | 6.8 | 18.2 |
| Spanish, ever use | 46.1 | 8.0 | 17.9 | 24.4 |
| csDMARDs [%] |  |  |  |  |
| Austrian | 82.0 | 19.1 | 47.4 | 51.5 |
| Norwegian, |  |  |  |  |
| Methotrexate | 56.1 | 6.5 | 41.2 | 41.3 |
| Other csDMARDs | 74.6 | 10.5 | 52.0 | 54.7 |
| Spanish | 87.0 | 32.4 | 74.5 | 64.9 |
| bDMARDs [%] |  |  |  |  |
| Austrian | 27.8 | 27.0 | 20.5 | 25.8 |
| Norwegian | 43.2 | 62.0 | 53.3 | 49.8 |
| Spanish | 40.4 | 47.4 | 41.7 | 43.2 |
| tsDMARDs [%] |  |  |  |  |
| Austrian | 6.0 | 0.0 | 12.8 | 5.5 |
| NSAIDs [%], regular or on request |  |  |  |  |
| Austrian | 41.0 | 74.8 | 62.8 | 58.1 |
| Norwegian | n.p. | n.p. | n.p. | n.p. |
| Spanish | 39.9 | 58.5 | 45.9 | 48.0 |

Abbreviations: ACPA, anti-citrullinated protein antibodies; bDMARD, biologic disease modifying drug; CRP, C-reactive protein; csDMARDs, conventional synthetic disease modifying drugs; ESR, erythrocyte sedimentation rate; HLA-B27, human leukocyte antigen B27; IJD, inflammatory joint disease; NSAID, non-steroidal anti-inflammatory drug; PsA, psoriatic arthritis; RA, rheumatoid arthritis; RF, rheumatoid factor; SpA, spondylarthritis; tsDMARD, targeted synthetic disease modifying drug. n.p., data not provided.
