# Supplementary material for: Cardiovascular Risks and Risk Stratification in Inflammatory Joint Diseases: A Cross-Sectional Study
Source: Front Med (Lausanne). 2022 Feb 22;9:786776. doi: 10.3389/fmed.2022.786776 (PMC8904360; doi:10.3389/fmed.2022.786776)
Supplement: Supplementary file 3 [file Table_3.DOCX]

**Supplementary Table S3. Patients’ disease characteristics and treatments according to cardiovascular risk group.**

|  | Low-risk  n=41 | Moderate-risk  n=10 | High-risk  n=17 | Very high-risk  n=14 | p |
| --- | --- | --- | --- | --- | --- |
| CRP [mg/dl] | 0.16 (0.33) | 0.25 (0.53) | 0.47 (0.65) | 0.36 (0.58) | 0.299* |
| ESR [mm/h] | 8 (13) | 10 (20) | 15 (16) | 16 (25) | 0.157* |
| Disease duration [years] | 12.4 (14.6) | 13.2 (18.3) | 14.1 (29.2) | 15.8 (22.2) | 0.646* |
| Median CS [mg/day] | 0 (0) | 0 (0) | 0 (1) | 0 (2) | 0.391* |
| csDMARDs [%] | 51.2 | 70.0 | 64.7 | 64.3 | 0.593^o^ |
| tsDMARDs [%] | 3.0 | 20.0 | 17.6 | 0.0 | 0.242^o^ |
| bDMARDs [%] | 46.3 | 30.0 | 11.8 | 35.7 | 0.092^o^ |
| NSAIDs |  |  |  |  |  |
| on request [%] | 48.8 | 20.0 | 35.3 | 50.0 | 0.332^o^ |
| regular [%] | 24.4 | 10.0 | 23.5 | 0.0 | 0.177^o^ |

Abbreviations: CRP, C-reactive protein; CS, dose of methylprednisolone; csDMARD, conventional synthetic disease modifying drug; ESR, erythrocyte sedimentation rate; IJD, inflammatory joint disease; NIRD, non-inflammatory rheumatic disease; NSAIDs, non-steroidal anti-inflammatory drugs; PsA, psoriatic arthritis; PsO, psoriatic disease; RA, rheumatoid arthritis; RF, rheumatoid factor; SpA, spondylarthritis; tsDMARD, targeted synthetic disease modifying drug. *- Kruskal-Wallis Test; ^o^ - Chi-squared test. All values are medians with interquartile ranges, if not specified otherwise.
