# Supplementary material for: Cardiovascular Risks and Risk Stratification in Inflammatory Joint Diseases: A Cross-Sectional Study
Source: Front Med (Lausanne). 2022 Feb 22;9:786776. doi: 10.3389/fmed.2022.786776 (PMC8904360; doi:10.3389/fmed.2022.786776)

Supplementary Figure S1. (A) Risks for 10-year fatal cardiovascular disease calculated with the original SCORE and (B) risks for 10-year fatal and non-fatal cardiovascular disease calculated with SCORE2 in patients with rheumatoid arthritis (RA) both without and with use of 1.5x multiplier according to EULAR), spondyloarthritis (SpA) and psoriatic arthritis (PsA aged 40 to 65 years - without diabetes mellitus, established cardiovascular disease, blood pressure ≥ 180/110 mmHg and total cholesterol > 310 mg/dL. (☐ low- to moderate, ■ high and ■ very high risk; data given in percentages).

Abbreviation: mRA, modified risk for rheumatoid arthritis with 1.5x multiplier.

A)

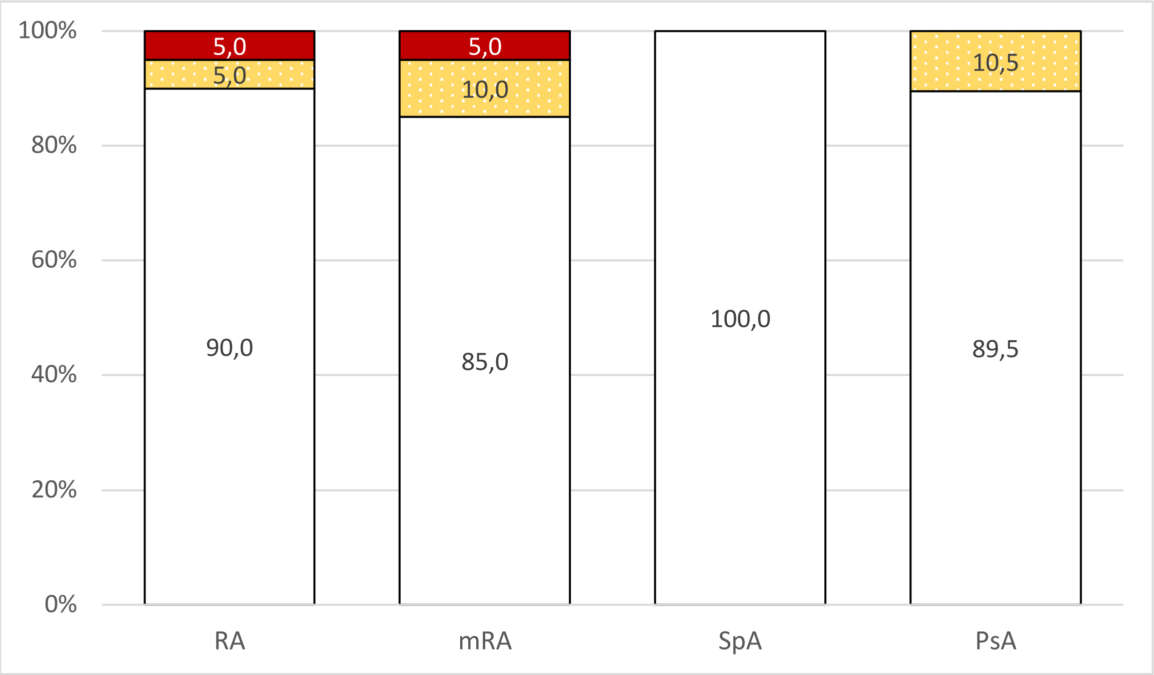

B)

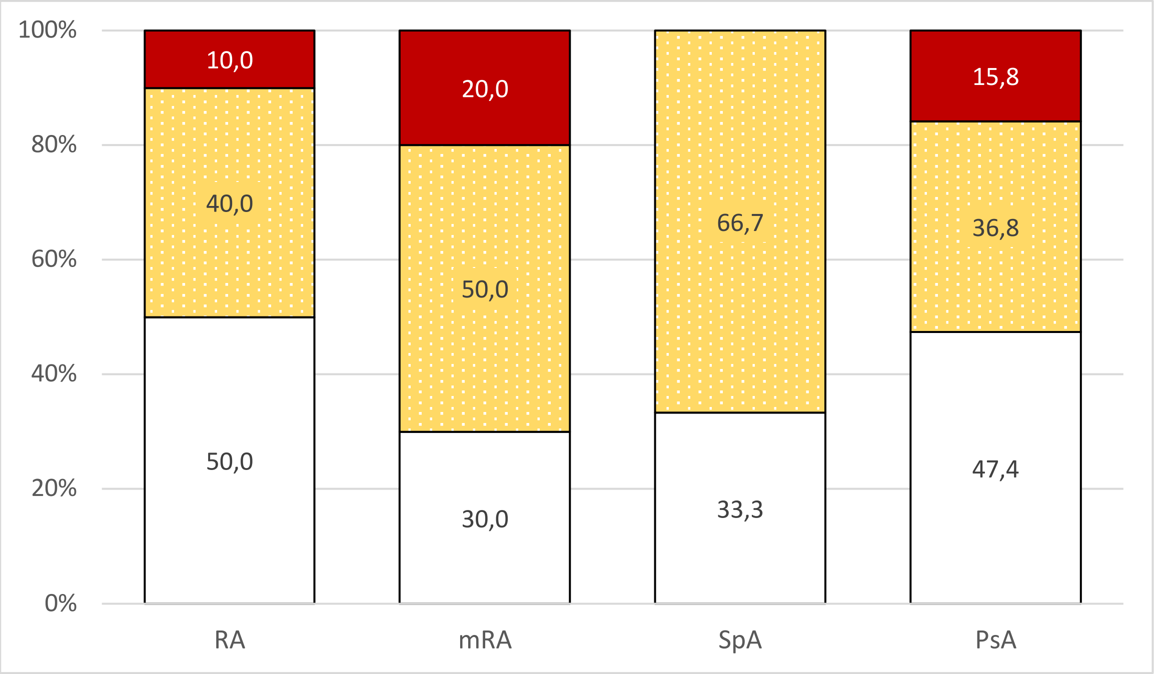

Supplement: Supplementary file 4 [file Image_1.pdf]
